# Relationship between *anaerobic* capacity estimated using a single effort and 30-s tethered running outcomes

**DOI:** 10.1371/journal.pone.0172032

**Published:** 2017-02-09

**Authors:** Alessandro Moura Zagatto, Willian Eiji Miyagi, Filipe Antônio de Barros Sousa, Claudio Alexandre Gobatto

**Affiliations:** 1 São Paulo State University (Unesp), School of Sciences, Laboratory of Physiology and Sports Performance (LAFIDE), Bauru-SP, Brazil; 2 Post-Graduate Program in Movement Sciences, São Paulo State University (Unesp), Institute of Biosciences, Rio Claro–SP, Brazil; 3 Campinas State University (UNICAMP), School of Applied Sciences, Limeira-SP, Brazil; University e-Campus, ITALY

## Abstract

The purpose of the current study was to investigate the relationship between alternative anaerobic capacity method (MAOD_ALT_) and a 30-s all-out tethered running test. Fourteen male recreational endurance runners underwent a graded exercise test, a supramaximal exhaustive effort and a 30-s all-out test on different days, interspaced by 48h. After verification of data normality (Shapiro-Wilk test), the Pearson’s correlation test was used to verify the association between the anaerobic estimates from the MAOD_ALT_ and the 30-s all-out tethered running outputs. Absolute MAOD_ALT_ was correlated with mean power (*r* = 0.58; *P* = 0.03), total work (*r* = 0.57; *P* = 0.03), and mean force (*r* = 0.79; *P* = 0.001). In addition, energy from the glycolytic pathway (E_[La_^-^_]_) was correlated with mean power (*r* = 0.58; *P* = 0.03). Significant correlations were also found at each 5s interval between absolute MAOD_ALT_ and force values (*r* between 0.75 and 0.84), and between force values and E_[La_^-^_]_ (*r* between 0.73 to 0.80). In conclusion, the associations between absolute MAOD_ALT_ and the mechanical outputs from the 30-s all-out tethered running test evidenced the importance of the *anaerobic* capacity for maintaining force during the course of time in short efforts.

## Introduction

Maximal accumulated oxygen deficit (MAOD) has been widely used as an *anaerobic* capacity estimative [1–4], i.e., the amount of energy that can be resynthesized by the phosphagen and glycolysis metabolism pathways. *Anaerobic* capacity has been well related to several exercise modes with a high-intensity and relatively short duration [5].

Some studies have described the effective use of the blood lactate response (i.e., post-exercise minus resting values) to estimate the oxygen equivalent corresponding to the glycolytic metabolic pathway [6–8] and the fast phase of excessive post-exercise oxygen consumption (EPOC_FAST_) to estimate the equivalent of oxygen corresponding to the phosphagen metabolic pathway [7,8]. In this way, some authors have reported that the sum of both equivalents of oxygen from the phosphagen and glycolytic metabolic pathways allow assessment of the *anaerobic* capacity in a single supramaximal exhaustive effort, denominated by authors of the alternative MAOD method (MAOD_ALT_) [9–14]. Recently Zagatto et al. [13] reported the validity, reliability and reproducibility of the MAOD_ALT_ for estimating the *anaerobic* capacity in treadmill running, evidencing that exercise intensity at 115% of maximal oxygen uptake (iVO_2max_) is the greatest intensity for MAOD_ALT_ determination. In addition, this same group of authors recently reported that MAOD_ALT_ assessed in treadmill running can be considered a sensitive enough procedure to distinguish the *anaerobic* capacity in individuals with different training levels (untrained, moderately active, recreational endurance runners and elite rugby sevens players) [15]

This method has a better practical application than the conventional MAOD method, which is basically assessed through oxygen uptake (VO_2_), measured in several submaximal and supramaximal trials [2], precluding its wide use in the training routine. In addition to assessment of *anaerobic* capacity, another advantage of the MAOD_ALT_ is the possibility of estimating the maximal energy contributions from the glycolytic and phosphagen metabolism pathways [9], a distinction that is unable to be performed using conventional MAOD.

Despite MAOD_ALT_ being easy to estimate regarding time required, it is a recent method that requires further investigation, principally comparing it with other scientifically accepted procedures, such as the 30-s Wingate anaerobic test [16].

The 30-s Wingate Anaerobic test is a widely used test to measure the *anaerobic* metabolism; it has been recognized that the peak power and mean power outputs measured during a 30-s Wingate Anaerobic test must represent the breakdown of phosphocreatine and muscle glycogen depletion, respectively [17,18]. In this way, the 30-s Wingate Anaerobic test has also been used to validate other *anaerobic* tests [5,9]. Recently, Bertuzzi et al. [9] described, in nine physical education students, significant correlations between MAOD_ALT_ measured in cycling with peak power (*r* = 0.78) and mean power (*r* = 0.79) from a 30-s Wingate *Anaerobic* test. Furthermore, the authors reported that the glycolytic metabolism pathway was correlated with mean power (*r* = 0.71) and the phosphagen metabolism pathway with peak power (*r* = 0.72) from the 30-s Wingate Anaerobic test [9]. Despite the relevant findings, the use of a 30-s Wingate *Anaerobic* test, such as reported by Bertuzzi et al. [9], is restricted to cycle ergometer and cannot be transferred to running, which is the most common locomotion mode performed by humans in sport, in addition to which, the cited study had a low sample size consisting of physical education students.

Recent studies evidenced higher correlations between sprint running performance and peak and mean power measured in running efforts, rather than on a cycle ergometer [1,16].

The running anaerobic sprint test was considered as an adaptation of the 30-s Wingate *Anaerobic* test for running [16,19] considering Newton's second law (force = mass x acceleration), but the use of body mass to calculate the force (i.e., force = body mass x distance/ time^2^) in horizontal running is the main limitation to assessing running power using this procedure. Therefore, the application of an all-out tethered run measuring the horizontal force may be more appropriate [1,20–23], and it can be used to investigate the association of MAOD_ALT_ and a 30-s maximal effort such as performed by Bertuzzi et al. [9] in cycling.

Thus, the purpose of the current study was to investigate the relationship between MAOD_ALT_ and parameters measured in a 30-s all-out tethered running test. Based on recent findings of Bertuzzi et al. [9] and studies that verified significant correlations between MAOD_ALT_ and the 30-s Wingate Anaerobic test, we hypothesized that MAOD_ALT_ would be significantly correlated with the 30-s all-out tethered running test outputs, chiefly regarding mean values.

In addition, such as previously investigations have reported that the MAOD_ALT_ is similar to conventional MAOD [13,14], the current study advances in literature [10,12–14,24] using only the MAOD_ALT_ method and assuming it as an valid method to estimate the *anaerobic* capacity [9,13,14,25].

## Materials and methods

### Subjects

The sample size was based on a sample calculation taking into consideration a significant correlation between MAOD_ALT_ and power output from the Wingate test of 0.79 [9] and a test power of 95%, resulting in a minimum sample size of eleven participants (software G*Power 3.0.10, Franz Faul, Germany).

Fourteen male recreational endurance runners were recruited for this study (mean±SD, age 29±4 years; height 177.0±6.1 cm; body mass 74.3±8.0 kg; body fat 16.2±3.8% and VO_2max_ 55.3±4.5 mL/kg/min). These subjects performed at least three running training sessions per week (weekly training volume ~40 km, at least three times per week), but were not professional athletes. The 10-km running times of the participants were between 40 and 60 min, which is a pace between 10 and 15 km/h. All subjects were familiarized with the experimental procedures and equipment and were instructed to eat the same individual light meal at least 2 hours before the tests, to maintain hydration habits, and to avoid additional sessions of hard physical activity and alcohol or caffeine ingestion during the experimental period.

### Inclusion and exclusion criteria

Participants were included in the study if they met the following inclusion criteria; a weekly training routine of at least 3 times per week, maximal oxygen uptake (VO_2max_) higher than 50 mL/kg/min and intensity at VO_2max_ (iVO_2max_) of at least 15 km/h, measured in a graded exercise test. Athletes who presented any kind of tendon, joint or skeletal muscle lesion were excluded from the sample, as were participants who took any dietary supplements during the 3 months prior to the start of the study.

The subject recruitment was performed via a direct approach to the coach of the recreational runner groups, who selected the runners according to the inclusion criteria required for the study. Subsequently, the runners were invited to participate in the study according to their personal and physical training availability.

The subjects were informed about the possible risks and benefits of the study prior to signing an informed consent, and all procedures were conducted respecting the declaration of Helsinki. The experimental procedures used in both studies, as well as the informed consent, were approved by the Research Ethics Committee of the University (Protocol number 645.784/2014).

### Experimental procedures

All exercise sessions were applied over two weeks, with the subjects performing four visits to the laboratory separated by a recovery interval of at least 48 hours. On the first visit the body composition was assessed by means of a whole-body dual-energy X-ray absorptiometry scan (Hologic QDR, Discovery, Bedford, USA) and each participant performed a familiarization session on the non-motorized treadmill. On the second visit a graded exercise test (GXT) was applied, and on the third and fourth visits a supramaximal exhaustive effort at 115% of the intensity associated with maximal oxygen uptake or a 30-s all-out tethered running test (30-s ATR) was performed in random order.

The GXT and supramaximal exhaustive effort test were performed on a motorized treadmill (ATL, Inbramed, Inbrasport, Porto Alegre, RS, Brazil) with a fixed treadmill incline of 1% [26,27], while the 30-s ATR was performed on a non-motorized treadmill adapted from a motorized model (Inbramed, Inbrasport, Porto Alegre, RS, Brazil), as used and detailed in recent studies [21,28]. To eliminate any influence of circadian variation, each subject completed all trials at the same time period of day in controlled environmental conditions regarding temperature (22.9 ± 1.3°C) and relative humidity (43.8 ± 6.3%). In all efforts, participants were verbally encouraged to perform maximally and wore a chest harness with the rope attached to the ceiling to ensure maximal effort without fall risk (except the 30-s ATR) [13,15]. Prior to each exercise trial, the subjects responded to the profile of mood states scale to measure their motivation for the effort. If a state of fatigue, low vigor, or stress was detected, a new date for the test was scheduled.

Prior to each effort, a warm-up lasting 5-min at 8 km/h was performed with the test starting four minutes after the end of the warm-up. Only for the 30-s ATR, in the warm-up, two sprints were added lasting 3-4s performed in the 3^rd^ and 4^th^ minutes.

#### Physiological analysis

Respiratory gas exchange and heart rate (HR) were collected breath-by-breath during all tests using a Cosmed Quark PFT gas analysis system (Quark PFT, Cosmed, Rome, Italy) coupled with a polar transmitter belt (T31, Polar Electro, Kempele, Finland). During the supramaximal effort, the VO_2_ was measured over the 10 minutes of rest before the warm-up (i.e., sitting in a chair), during the efforts, and for seven minutes after the end of the exercise, to assess the fast component of excess post-exercise oxygen consumption (EPOC_FAST_). Before each test, the gas analyzer was calibrated using an ambient air sample and a high-precision gas mixture (3.98% CO_2_ and 16.02% O_2_; White Martins^®^, Osasco, Brazil), whereas the spirometer was calibrated using a 3-liter syringe (Hans Rudolf, Kansas City, Missouri, USA), in accordance with the manufacturer’s instructions. After the removal of outliers to exclude discrepant breaths, breath-by-breath VO_2_ data were smoothed using a 5-second moving average and interpolated to give 1-second values (OriginPro 8.0; Origin Lab Corporation, Microcal, MA, USA) to enhance the underlying VO_2_ response characteristics [29].

To measure the blood lactate concentration ([La^-^]), blood samples were drawn from the earlobe (25 μL) after 10 minutes of rest and 3, 5 and 7 minutes after the end of exercise in the supramaximal test and the 30-s ATR, while in the GXT the blood samples were drawn only after the effort. Blood samples were stored at -20°C in tubes containing 50 micro-liters of sodium fluoride (1%) and later analyzed using an electrochemical lactate analyzer (Yellow Springs Instruments model 2300, Ohio, USA) to determine the [La^-^] (measurement error of ±2%).

#### Graded Exercise Test (GXT) to assess VO_2max_ and iVO_2max_

The GXT began at 8 km/h with stage increments of 1.5 km/h every 2 minutes until exhaustion, given voluntarily by the participant or by their inability to perform the effort at the pre-determined speed [10,13]. The GXT was based on the guidelines of Howley et al.[30] for VO_2max_ and designed to last 8–12 minutes. The Borg scale (6–20) [31] was used to assess the rate of perceived exertion (RPE) at the end of each stage of the GXT. VO_2_ was measured during the entire test and the highest VO_2_ average (i.e., VO_2_ average over the final 30s of each stage) was assumed as VO_2max_, considering the verification of a plateau in VO_2_ (variation in VO_2_<2.1 mL·kg^-1^·min^-1^ between the final and penultimate stage of exercise). Secondary criteria were: 1) maximal HR (HRmax) ≥ 90% of predicted HRmax [32]; 2) respiratory exchange ratio (RER) ≥ 1.10 and 3) peak lactate ≥ 8.0 mmol·L^-1^. The minimal exercise intensity at which the subject reached VO_2max_ was considered as iVO_2max_ [33]. If the final stage had not been completed, the iVO_2max_ was determined using the method proposed by Kuipers et al. [34] [iVO_2max_ = running speed of the final complete stage + (velocity increment after each stage × time sustained during the incomplete stage / total time of stage)].

#### 30-s All-out Tethered Running Test (30-s ATR)

The 30-s ATR was performed on a non-motorized treadmill (Inbramed, Inbrasport, Porto Alegre, RS, Brazil) and consisted of running in an all-out maximal effort for 30-s [23], with velocity and horizontal force measured by a high frequency (1000 Hz) signal acquisition system [21,28,35].

**Fig 1:**
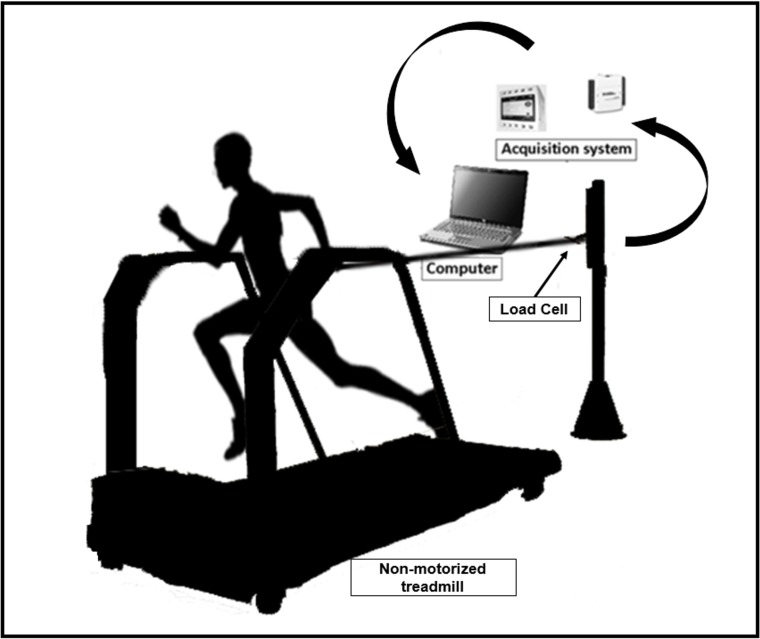
Schematic model for the 30-s all-out tethered running test on a non-motorized treadmill.

The signal acquisition system consisted of a strain gage (CSA/ZL-100, MK Control, Sao Paulo, Brazil), a portable amplifier (MKTC5-10, MK Control, Sao Paulo, Brazil) and a data acquisition module (NI USB-6009, National Instruments, Austin, USA). The strain gage was connected to the runner’s waist by an inextensible thread (2-m) and a nylon belt. This way, data of force was measured by the strain gages, while velocity was calculated as the first derivative of displacement, which was measured by a pulse sensor placed in the treadmill. Force and displacement signals were captured using specific software (LabView2012—National Instruments, Signal Express—Austin, Texas, EUA) and then transferred and analyzed using MatLab (MatLab^®^, MathWorks^tm^, USA) (Fig 1). The force and velocity data were synchronized each 1-ms for assessment of running power (i.e., multiplication of force and velocity during the effort). Next, 5-s averages were performed for determination of peak power (highest 5-s value), mean power (average of all 5-s values) and power decrement [36]. The peak power and mean power were presented in absolute and relative to body mass values. Likewise, the peak force, mean force, mean velocity and distance covered were also determined considering 5-s averages. Lastly, total work and the time to attain the maximal velocity were also assessed [28]. The power measurement in sprinting on a non-motorized treadmill measured by force transducers and goniometers has presented high test-retest reliability (r = 0.94; ICC = 0.90) [22]

#### Supramaximal exhaustive effort and assessment of MAOD_ALT_

The supramaximal exhaustive effort consisted of a maximal effort at 115% of iVO_2max_ [13]. The time to exhaustion was recorded. Energetic contributions from the phosphagen metabolic pathway (E_PCr_) and glycolytic metabolic pathway (E_[La_^-^_]_) were estimated during the test and the MAOD_ALT_ was assumed as the sum of both oxygen equivalents [3,7,8,10,11,14]. Zagatto et al. [13] reported high test and retest reliability for MAOD_ALT_ determined at 115% of iVO_2max_ during treadmill running (ICC = 0.87).

The E_[La_^-^_]_ amount of energy was estimated by subtracting resting blood lactate from post-exercise blood lactate concentration (Δ[La^-^]), considering a value of 1 mmol.L^-1^ to be equivalent to 3 mL O_2_/kg body mass.[7,8] The E_PCr_ contribution was considered to be the EPOC_FAST_ [6,7,13,37], which was estimated by multiplying the amplitude and the time constant of the fast component of a bi-exponential model (Eq 1) using OriginPro 8.0 software (OriginLab Corporation, Microcal, Massachusetts, USA) (Eq 2) [13,25,37,38].

$${\text{VO}}_{2(\text{t})}=\text{V}{\text{O}}_{2\text{baseline}}+{\text{A}}_{1}[{\text{e}}^{\text{-}(\text{t-}\delta )/\tau_{1}}]+{\text{A}}_{2}[{\text{e}}^{\text{-}(\text{t-}\delta )/\tau_{2}}]$$ Equation (1)

where VO_2(t)_ is the oxygen uptake at time t, VO_2baseline_ is the oxygen uptake at baseline, A is the amplitude, δ is the time delay and τ is the time constant. The numbers 1 and 2 after A represent the fast and slow components, respectively, and the EPOC_FAST_ was calculated by the product of A_1_ and τ_1_.

#### Statistical analysis

The data are presented as mean ± standard deviation (SD) and confidence interval of 95% (CI95%). Data normality was initially verified using the Shapiro-Wilk test allowing the use of parametric statistical analysis. The Pearson’s correlation test was used to verify the association between the MAOD_ALT_ value and the 30-s ATR outputs. The coefficient of correlation was classified as very weak to negligible (0 to 0.2), weak (0.2 to 0.4), moderate (0.4 to 0.7), strong (0.7 to 0.9), and very strong (0.9 to 1.0).[39] In all cases, a significance level of 5% was assumed. All statistical analysis was performed using the Statistical Package for Social Sciences (SPSS Inc. Released 2009. PASW Statistics for Windows, Version 18.0. Chicago, USA)

## Results

In the GXT, all subjects attained the criteria for assessment of VO_2max_, with [La^-^] peak values of 11.4 ± 2.1 mmol/L (CI95% = 10.1 − 12.6 mmol/L), heart rate of 188.7 ± 5.4 bpm (CI95% = 185.6 − 191.8 bpm) and respiratory exchange ratio of 1.16 ± 0.06 (CI95% = 1.13 − 1.19). Therefore, the VO_2max_ was 55.3 ± 4.5 mL/kg^/^min (CI95% = 52.6 −57.9 mL/kg^/^min) and the iVO_2max_ was 16.4 ± 0.9 km/h (CI95% = 16.0 − 17.1 km/h).

**Fig 2:**
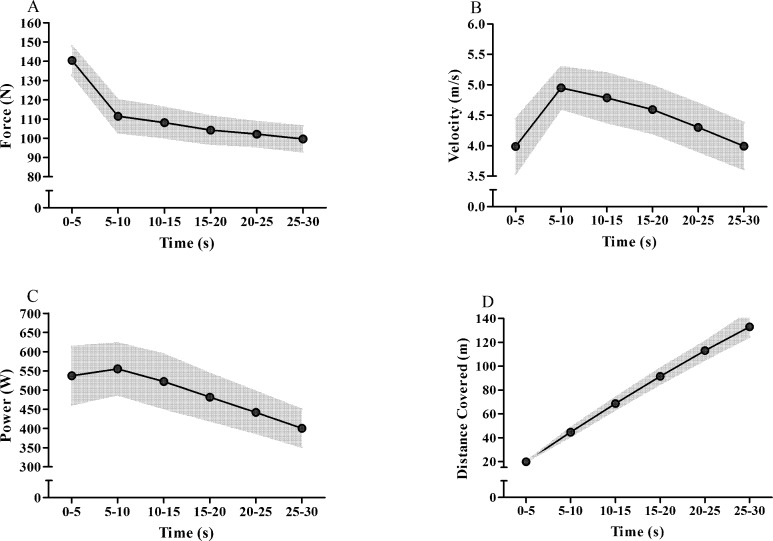
Values of force (A), velocity (B), power (C) and distance covered (D) during the 30-s all-out tethered running test averaged each 5-s of effort. The circles are the mean values and the dark gray area corresponds to the upper and lower standard deviations.

The mechanical variables measured during the 30-s ATR are shown in Table 1, whereas Fig 2 shows the behavior of force (2A), velocity (2B), power (2C) and distance covered (2D) measured over time (i.e., values measured as 5-s averages).

**Table 1:** Values of power, force, velocity power decrement, total distance covered, total work and time to attain the maximal velocity measured in the 30-s ATR. Results in Mean ± SD and CI95%.

Variables	Mean ± SD	95%IC
Peak Power (W)	568.4 ± 61.9	532.7 − 604.2
Mean Power (W)	489.9 ± 51.3	460.2 − 519.5
Peak Power (W/kg)	7.7 ± 0.8	7.3 − 8.1
Mean Power (W/kg)	6.6 ± 0.6	6.3 − 6.9
Peak Force (N)	140.5 ± 7.5	136.2 − 144.8
Mean Force (N)	111.0 ± 6.7	107.1 − 114.9
Peak Velocity (m/s)	4.98 ± 0.30	4.80 − 5.15
Mean Velocity (m/s)	4.44 ± 0.28	4.27 − 4.60
Power Decrement (%)	13.6 ± 6.3	10.0 − 17.2
Total Distance covered (m)	133.0 ± 8.6	128.0 − 137.9
Total work (kJ)	13.7 ± 1.5	12.8 − 14.5
Time to attain the maximal velocity (s)	9.6 ± 5.4	6.5 − 12.7

The exercise intensity in the supramaximal exhaustive test of 115% of iVO_2max_ corresponded to 19.1 ± 1.1 km/h (CI95% = 18.4 − 19.7 km/h) and the time to exhaustion was 109.5 ± 29.8 s (CI95% = 92.3 − 126.7 s). The peak [La^-^] was 11.6±2.0 mmol/L (CI95% = 10.5 − 12.8 mmol/L), with Δ[La^-^] corresponding to 10.2±1.9 mmol/L (CI95% = 9.2 − 11.4 mmol/L), whereas the A_1_ and τ1 from the EPOC_FAST_ were 21.3±2.4 mL/kg/min (CI95% = 20.0 − 22.7 mL/kg/min) and 1.00±0.13 min (CI95% = 0.92 − 1.08 min), respectively. Concerning the oxygen equivalents estimated from the glycolytic and phosphagen metabolic pathways during this test, the E_[La_^-^_]_ corresponded to 2.29 ± 0.52 L (CI95% = 2.00 − 2.59 L) (30.8±5.7 mL/kg; CI95% = 27.5 − 34.1 mL/kg), whereas the E_PCr_ was 1.58 ± 0.26 L (CI95% = 1.43 − 1.73 L) (21.2±2.5 mL/kg; CI95% = 19.8 − 22.7 mL/kg). Therefore, the absolute MAOD_ALT_ corresponded to 3.87 ± 0.71 L (CI95% = 3.46 − 4.28 L) and MAOD_ALT_ relative to body mass was 52.0 ± 6.8 mL/kg (CI95% = 48.0 − 55.9 mL/kg) (S1 Dataset).

Table 2 shows the coefficient of correlations between the 30-s ATR outputs and MAOD_ALT_. Absolute MAOD_ALT_ was statistically moderately correlated with mean power (*r* = 0.58, CI95% = 0.08 − 0.85; *P* = 0.03) and total work (*r* = 0.57, CI95% = 0.06 − 0.85; *P* = 0.03), and strongly correlated with mean force (*r* = 0.79, CI95% = 0.45 − 0.93; *P* = 0.001), however no significant correlation for MAOD_ALT_ was found when presented relative to body mass. In addition, the E_[La-]_ was moderately correlated with mean power (*r* = 0.58, CI95% = 0.07 − 0.85; *P* = 0.03), but a weak and non-significant correlation was found between E_PCr_ and peak power (*r* = 0.33, CI95% = -0.59 − 0.47; *P* = 0.25) and other outcomes.

**Table 2:** Coefficient of Correlation between the 30-s ATR outputs and MAOD_ALT_. Values in *r* (CI95%).

Variables	MAOD_ALT_ (L)	MAOD_ALT_ (mL·kg^-1^)
Peak power (W)	0.38(-0.19 − 0.76)	0.13(-0.52 − 0.54)
Mean Power (W)	0.58*(0.08 − 0.85)	0.34(-0.30 − 0.70)
Peak Power (W/kg)	-0.34(-0.74 − 0.23)	-0.19(-0.66 − 0.37)
Mean Power (W/ kg)	-0.16(0.64 − 0.41)	0.10(-0.46 − 0.60)
Peak Force (N)	0.31(-0.26 − 0.72)	-0.09(-0.59 − 0.46)
Mean Force (N)	0.79^¥^(0.45 − 0.93)	0.40(-0.17 − 0.77)
Peak Velocity (m/s)	0.07(-0.48 − 0.58)	-0.13(-0.62 − 0.43)
Mean Velocity (m/s)	0.18(-0.38 − 0.65)	0.05(-0.50 − 0.56)
Power decrement (%)	-0.27(-0.70 − 0.30)	-0.39(-0.76 − 0.18)
Total Distance Covered (m)	0.17(-0.40 − 0.64)	0.05(-0.49 − 0.57)
Total Work (kJ)	0.57*(0.06 − 0.85)	0.25(-0.32 − 0.69)
Time to attain the maximal velocity (s)	0.30(-0.26 − 0.72)	0.48(-0.07 − 0.81)

* *P* < 0.05

^¥^ *P* < 0.01.

**Fig 3:**
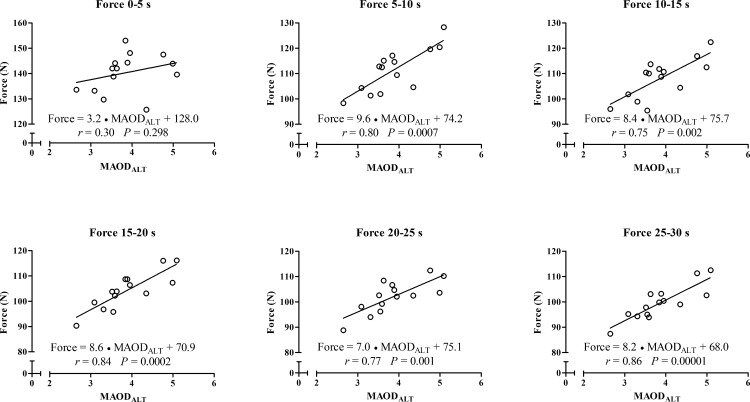
Linear regression and coefficient of correlation between force measured each 5-s during the 30-s ATR with MAOD_ALT_.

**Fig 4:**
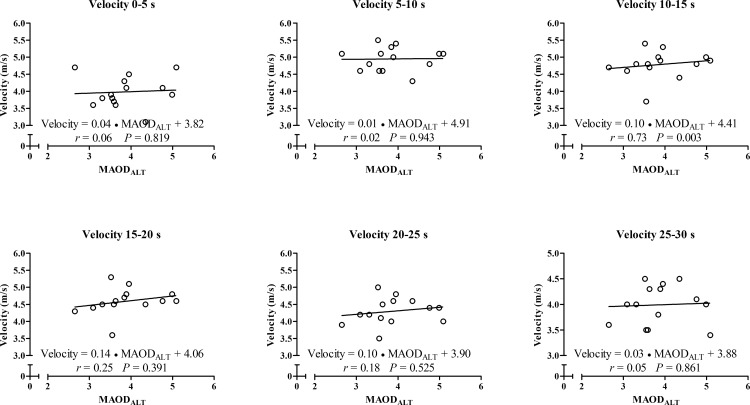
Linear regression and coefficient of correlation between velocity measured each 5-s during the 30-s ATR with MAOD_ALT_.

**Fig 5:**
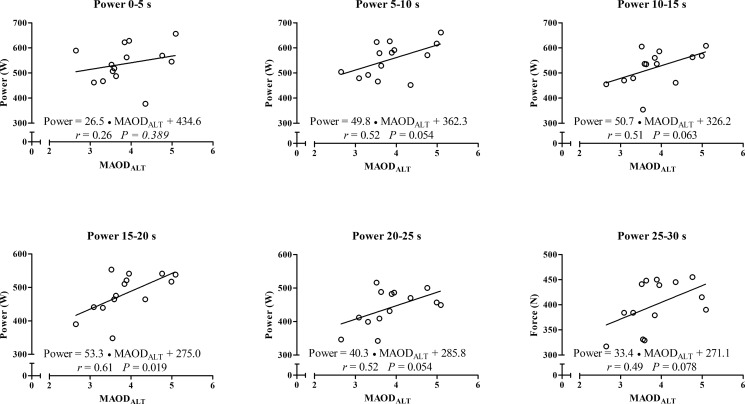
Linear regression and coefficient of correlation between power measured each 5-s during the 30-s ATR with MAOD_ALT_.

**Fig 6:**
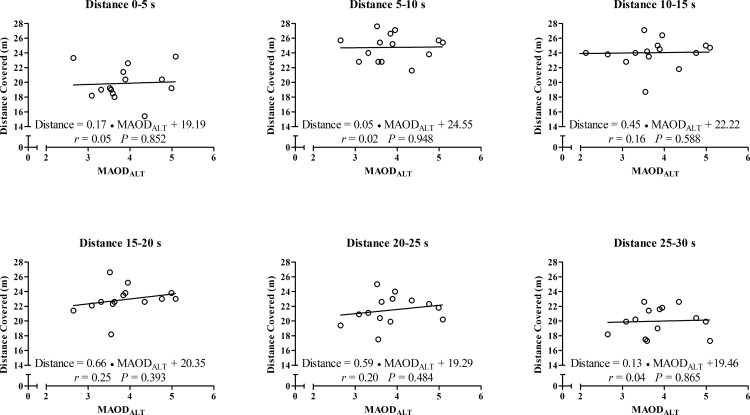
Linear regression and coefficient of correlation between distances covered measured each 5-s during the 30-s ATR with MAOD_ALT_.

In addition, for the correlations between peak and mean outputs from the 30s- ATR, the MAOD_ALT_ was also strongly correlated with force values at 5–10 s until 25–30 s (Fig 3) and moderately correlated with the power and velocity values at 15–20 s (Fig 4 and Fig 5, respectively), but not with distances covered (Fig 6). Force values at 5–10 s until 25–30 s were also strongly correlated with E_[La_^-^_]_ (*r* = 0.53 to 0.63; *p* < 0.01) and E_PCr_ (*r* = 0.60 to 0.74; *P* ≤ 0.025), while the power values at 5–10 s until 20–25 s were also moderately correlated with E_[La-]_ (*r* = 0.54 to 0.62; *P* ≤ 0.049). However, there was no significant correlation between anaerobic energy estimation and values of velocity or distance covered.

## Discussion

The main findings of this study were the MAOD_ALT_ correlations with mean power, mean force and total work, and, principally, the correlations of MAOD_ALT_, E_PCr_ and E_[La_^-^_]_ with force values from the 5–10 s to 25-30s periods in the 30-s ATR and E_[La_^-^_]_ with power values, confirming our initial hypothesis.

The conventional MAOD has been significantly correlated with 30-s Wingate test outputs [5]. However, the conventional MAOD is unable to distinguish both the glycolytic and phosphagen metabolic pathways in the same test, besides which the conventional MAOD requires a huge amount of time to assess the *anaerobic* capacity in a robust procedure without overestimating the energy demand during the supramaximal effort [9,40].

More recently Bertuzzi et al.[9] also described associations between MAOD_ALT_ and 30-s Wingate test outputs. However, since these authors used the MAOD_ALT_ method, they also reported significant correlations of the phosphagen and glycolytic metabolism pathways with peak power and mean power, respectively, for the first time. In our findings the E_PCr_ was weak and non-significantly correlated with peak power (*r* = 0.33, CI95% = -0.59 − 0.47; *P* = 0.25). As the 30-s ATR was performed on a non-motorized treadmill and beginning with the treadmill belt stopped, the improvement and attainment of peak value for the force was faster than the response of velocity and power, therefore delaying the peak power value to near 10 seconds (see Fig 2A, 2B and 2C), instead the peak value occurred in the first seconds of exercise such as observed during the Wingate test. Mangine et al. [23] reported similar findings, with the peak velocity being attained at 7.22±3.77 s during a 30-s tethered running test. Considering that the phosphagen metabolism is predominantly activated during the first seconds of effort, the E_PCr_ was mainly correlated with force values (force values at 5–10 s until 25–30 s; *r* = 0.60 to 0.74; *P* ≤ 0.025) and weakly correlated with power values, which were more correlated with E_[La_^-^_]_. The delayed response of power during the 30-s ATR is the main factor to explain the difference between the findings of our study and those of Bertuzzi et al. [14] concerning the association between E_PCr_ and peak power during a 30-s all-out effort.

The current study is the first to investigate the associations between MAOD_ALT_ and a 30-s all-out test in running. In addition, the assessment of power using a high frequency signal acquisition system to measure the horizontal force and velocity improves the quality of the mechanical work measurements during this test, which is a novelty of this study.

All studies that have compared *anaerobic* parameters with a 30-s Wingate test have used the mean power, peak power, fatigue index and total work as the main outputs [5,9]. The data from the present study reported correlations of MAOD_ALT_ with mean power, mean force and total work, which are parameters that can be considered as the overall *anaerobic* metabolism [5,41]. All-out tethered running is considered a good test to assess the specific sprint-running *anaerobic* power [42], but the lack of significant correlations between MAOD_ALT_ and peak power values can be explained also due to power not indicating capacity and consequently the peak power from the 30-s Wingate test (similar to the 30-s ATR) is not a good predictor of *anaerobic* capacity, as also reported by Minahan et al. [43]. Minahan et al. [43] demonstrated that there is no significant relationship between peak power from the 30-s Wingate *anaerobic* test and the conventional MAOD in cycling, similar to the findings of the present study (r = 0.38 for MAOD_ALT_ in L and 0.13 for MAOD_ALT_ relative to body mass; Table 2).

In a similar study, Andrade et al. [19] also reinforced these findings, reporting that power outputs from a running-based *anaerobic* sprint test (i.e., adaptation of the 30-s Wingate *anaerobic* test for running) are not correlated with conventional MAOD. On the other hand, the duration of the all-out test results in a high influence of *anaerobic* capacity on the total (work) and mean (force and power) mechanical parameters, as suggested by the significant correlations presented here.

A novelty of the current investigation is the strong and significant correlations of MAOD_ALT_, E_[La-]_ and E_PCr_ with force values in the course of time (Fig 3), and the correlation of E_[La_^-^_]_ with power values from the 5–10 s to 20–25 s periods during the 30-s ATR. These correlations describe the importance of *anaerobic* capacity estimated through MAOD_ALT_ and of the E_[La_^-^_]_ and E_PCr_ to produce high force over time during the test. Subjects with higher *anaerobic* capacity produce higher force values during almost the entire test, and the glycolytic metabolism has a moderate influence on power values. In addition, during a tethered running effort the force generation seems be higher than during a free-running effort such as a running-based *anaerobic* sprint test, explaining the significant correlation found in the current study in comparison to the study of Andrade et al. [19].

Moreover, several studies have reported that horizontal force during sprinting is the strongest predictor of acceleration [44] and short distance performance (i.e., 100-m) [45], highlighting the relationship between MAOD_ALT_ and horizontal force outcomes found in the current study.

### Limitations of study

A limitation of the MAOD_ALT_ method which should be considered is the fact that the E_[La_^-^_]_ could be underestimated as the lactate production is dependent on active body mass during effort and also a portion of the lactate produced by muscles can be oxidized during exercise. In addition, the EPOC_FAST_ can also be affected by some factors such as caffeine, which can hasten the oxidative metabolism and decrease the time constant [46]. Moreover, a limitation of the present study is the fact that MAOD_ALT_ was not compared with the conventional MAOD method; however, this was based on the findings of Bertuzzi et al. [14], Zagatto et al. [13] and Miyagi et al. [25], who reported the similarity between *anaerobic* estimated using the MAOD_ALT_ and MAOD. In addition, in the current study we used a sample size of 14 runners, which is considered a satisfactory sample size based on a statistical power of 95%, however, this must also be considered as a limitation. Finally, further investigation of MAOD_ALT_ using sprinters is recommended.

## Conclusion

In conclusion, the absolute MAOD_ALT_ was moderately associated with mean power and total work, and it was strongly associated with mean force from a 30-s all-out tethered running test, and, principally, with force behavior over time during the test, evidencing the importance of *anaerobic* capacity to maintain force over the course of time in short efforts.

## Supporting information

S1 Dataset Dataset. (XLSX)
